# Separation and Detection of Catechins and Epicatechins in Shanxi Aged Vinegar Using Solid-Phase Extraction and Hydrophobic Deep Eutectic Solvents Combined with HPLC

**DOI:** 10.3390/molecules29102344

**Published:** 2024-05-16

**Authors:** Baoqing Bai, Dan Shen, Siyuan Meng, Yanli Guo, Bin Feng, Tao Bo, Jinhua Zhang, Yukun Yang, Sanhong Fan

**Affiliations:** 1 School of Life Science, Shanxi University, Taiyuan 030006, China; baoqingbai@sxu.edu.cn (B.B.); 202123117011@email.sxu.edu.cn (D.S.); 202223109012@email.sxu.edu.cn (S.M.); 202223117005@email.sxu.edu.cn (Y.G.); botao@sxu.edu.cn (T.B.); ever840605@sxu.edu.cn (J.Z.); 2 Xinghuacun College, Shanxi University, Taiyuan 030006, China; 3 Inspection and Testing Center of Shanxi Province, Taiyuan 030031, China; fbily238@163.com; 4 Shanxi Key Laboratory of Food and Drug Safety Prevention and Control, Taiyuan 030031, China; 5 Key Laboratory of Chemical Biology and Molecular Engineering of Ministry of Education, Institute of Biotechnology, Shanxi University, Taiyuan 030006, China

**Keywords:** hydrophobic deep eutectic solvent, vortex-assisted solid phase extraction, catechin, epicatechin, Shanxi aged vinegar

## Abstract

This research presents a new, eco-friendly, and swift method combining solid-phase extraction and hydrophobic deep eutectic solvents (DES) with high-performance liquid chromatography (SPE-DES-HPLC) for extracting and quantifying catechin and epicatechin in Shanxi aged vinegar (SAV). The parameters, such as the elution solvent type, the XAD-2 macroporous resin dosage, the DES ratio, the DES volume, the adsorption time, and the desorption time, were optimized via a one-way experiment. A central composite design using the Box–Behnken methodology was employed to investigate the effects of various factors, including 17 experimental runs and the construction of three-dimensional response surface plots to identify the optimal conditions. The results show that the optimal conditions were an HDES (tetraethylammonium chloride and octanoic acid) ratio of 1:3, an XAD-2 macroporous resin dosage of 188 mg, and an adsorption time of 11 min. Under these optimal conditions, the coefficients of determination of the method were greater than or equal to 0.9917, the precision was less than 5%, and the recoveries ranged from 98.8% to 118.8%. The environmentally friendly nature of the analytical process and sample preparation was assessed via the Analytical Eco-Scale and AGREE, demonstrating that this method is a practical and eco-friendly alternative to conventional determination techniques. In summary, this innovative approach offers a solid foundation for the assessment of flavanol compounds present in SAV samples.

## 1. Introduction

**Figure 1:**
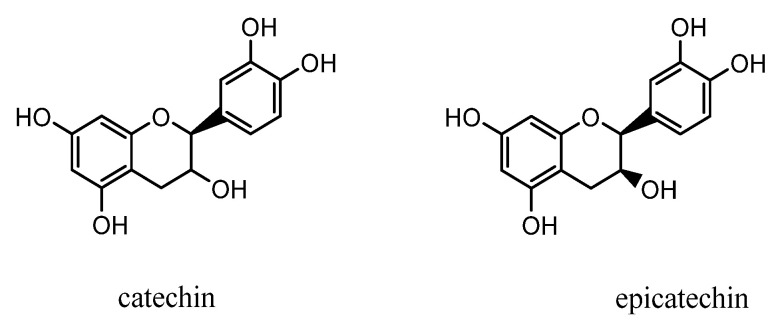
The structure of catechins and epicatechins.

In recent years, nutritional research has focused on the study of polyphenolic active compounds, which are widely found in a variety of foods and beverages, in order to elucidate their beneficial effects on human health. Flavanols are a major class of polyphenolic compounds, with catechin and epicatechin being two important monomeric compounds [1]. Numerous studies show that catechin, which is found in the human diet, is essential for the prevention of degenerative diseases and certain types of cancers [2]. Additionally, catechins provide health benefits, including hypolipidemic, hypoglycemic, and free radical scavenging properties [3]. Epicatechins have also been proven to enhance vascular function and insulin sensitivity, as well as decreasing blood pressure and platelet reactivity [4,5,6,7]. The strong antioxidant activity properties of the catechins and epicatechins are due to their polyphenolic structure (Figure 1) [8]. Shanxi aged vinegar (SAV) is one of China’s four renowned vinegars, brewed using the traditional solid-state fermentation process, and is well liked by consumers for its distinctive taste, wealth of nutrients, and diverse functional components [9,10]. SAV is considered to have health benefit due to its high polyphenol content, highlighting the relationship between it and properties that promote human health [11]. Therefore, the determination of the contents of catechins and epicatechins in SAV is crucial to further investigate their physiological activities as health factors of SAV.

In the last two decades, a number of pretreatment techniques have been developed for the extraction and purification of phenolic compounds from sample matrices. These methods comprise liquid–liquid extraction (LLE) [12], dispersive liquid–liquid microextraction (DLLME) [13], solid-phase extraction (SPE) [14], and supercritical fluid extraction (SFE) [15,16]. SPE is an advanced extraction method with the advantages of simplicity, reproducibility, low reagent usage, and high sample recovery. This technique is used to pre-treat food, environmental, and biological samples by increasing the concentration of trace amounts of specific compounds [17,18]. The SPE technique offers a broad choice of adsorbents including macroporous polymer resins, silica gel, alumina, and activated carbon [19]. Resin adsorption is the preferred adsorbent due to its simplicity in design, ease of regeneration, high adsorption rate, and high adsorption capacity compared to traditional adsorbents such as silica gel, alumina, and activated carbon, and is considered one of the most effective methods for the enrichment and recovery of secondary metabolites from polyphenolic plants [20,21,22,23]. Furthermore, XAD-2 macroporous adsorbent resins are edible [24], and although they have been thoroughly investigated as an eco-friendly extractant, there are scant reports available on the extraction of polyphenols from vinegar.

Although SPE enhances extraction efficiency to a certain degree, it presents a challenge due to the use of substantial quantities of toxic organic solvents for elution. In 2000, the notion of green analytical chemistry (GAC) was introduced [25]. Its primary aim is to reduce the adverse impacts of analytical techniques on the environment or researchers and to replace or diminish the usage of dangerous compounds, which has garnered more scientific attention. Deep eutectic solvents (DESs) are regarded as an innovative and environmentally friendly solvent, offering multiple advantages in the field of GAC [26,27]. Hydrophobic deep eutectic solvents (HDESs) are specially designed solvents with low or negligible water miscibility, low vapor pressure, a wide liquid range, low flammability, and high solvation ability [28]. After 2015, the synthesis and application of hydrophobic deep eutectic solvents (HDESs) commenced [28]. This was due to the distinctive properties of HDESs, which render them excellent extractant for a multitude of targets. Additionally, HDESs align with the tenets of green analytical chemistry, making them promising substitutes for traditional organic solvents in sample preparation. So far, HDESs have been successfully applied to the purification of water [29], polypropylene acetate in Ginkgo biloba [30], and artemisinin in Artemisia absinthium leaves [31].

In this study, an SPE-DES method was developed using XAD-2 macroporous resin as a new adsorbent for the extraction of flavanols from Shanxi aged vinegar. The key process parameters such as the elution solvent type, XAD-2 macroporous resin dosage, HDES ratio, HDES volume, adsorption time, and desorption time were optimized using a single factor. The main influencing factors were screened via the Placket–Burman design (PBD) and further optimized by means of the Box–Behnken design (BBD) method to obtain the optimal extraction process of flavanols from Shanxi aged vinegar. In addition, this method was compared with some reference techniques and was found to be advantageous in terms of detection limit and sensitivity. Finally, two tools, Analytical Eco-Scale and AGREE, were used to evaluate the environmental friendliness of the method, which meets the requirements of green analytical chemistry.

## 2. Results and Discussion

### 2.1. Optimization of the Extraction Procedure

#### 2.1.1. Elution Solvent

**Figure 2:**
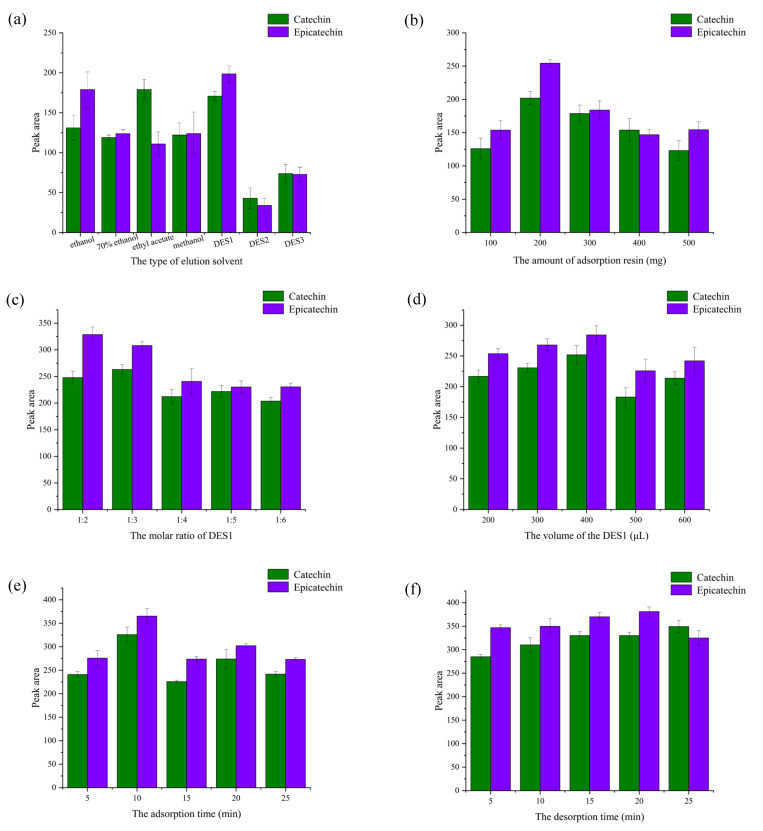
The results of the effect of the types of elution solvents (**a**), the amount of adsorption resin (**b**), the molar ratio of DES1 (**c**), the volume of the DES1 (**d**), adsorption time (**e**), and desorption time (**f**).

The selection of appropriate eluents is a key step in SPE processing. In this study, ethanol, 70% ethanol, ethyl acetate, methanol, and DES (DES1: tetraethylammonium chloride: octanoic acid; DES2: tetrabutylammonium chloride: octanoic acid; DES3: choline chloride: acetic acid, with molar ratio of 1:2) were used for elution of the flavanols compounds. DES1 eluted the highest peak area for epicatechin, and the highest peak area for catechin was eluted with ethyl acetate, followed by DES3 (Figure 2a). This was probably due to the polarity compatibility of the analyte with DES1, which causes maximum interaction between flavanols and DES1 [32]. Due to its superior elution performance, DES1 was selected as the elution solvent for the subsequent procedures.

#### 2.1.2. The Amount of XAD-2 Macroporous Adsorption Resin

Another parameter affecting the extraction efficiency of the analyte is the amount of XAD-2 macroporous adsorption resin added. The peak area gradually increased when the XAD-2 macroporous adsorption resin content was increased from 100 mg to 200 mg (Figure 2b). However, the peak areas of the target compounds decreased when the XAD-2 macroporous adsorption resin content was further increased from 200 mg to 500 mg. It is possible that the excessive adsorbent causes the incomplete elution of target compounds, thus reducing the efficiency of solid phase extraction [33]. Therefore, the optimal amount of XAD-2 macroporous adsorption resin was determined to be 200 mg.

#### 2.1.3. Molar Ratio of HBA/HBD

For extraction efficiency, the molar ratio of HBA/HBD in the DES is critical. By changing the molar ratio between HBA and HBD, the viscosity, surface tension, and hydrogen bonding strength of DES can be adjusted [34]. As shown in Figure 2c, when the molar ratio of HBA/HBD in DES1 was 1:3, the peak areas reached their maximum value. This showed that HBA and HBD had the strongest binding force with the two active ingredients at the 1:3 molar ratio, being efficient and stable. Octanoic acid has excellent mobility and a high diffusion rate, thus reducing the viscosity of the solution and increasing the solubility of the target analyte in the solution. The peak areas of epicatechin gradually decreased as the molar ratio of octanoic acid increased from 1:3 to 1:6. This may be because octanoic acid reduces the number of hydrogen bonds between HBAs and HBDs, thereby reducing extraction efficiency [34]. In summary, the molar ratio of tetraethylammonium chloride to octanoic acid was found to be 1:3 for the best elution effect.

#### 2.1.4. Extraction Solvent Volume

To achieve high and stable extraction efficiencies, this study investigated the effect of different volumes of DES1 (200 μL, 300 μL, 400 μL, 500 μL, and 600 μL) on the extraction efficiency. As the volume of DES1 increased from 200 µL to 400 µL, the peak area of the target compounds increased (Figure 2d). This is likely due to insufficient extraction of the targeted compounds given the limited volume of DES1 [35]. The peak area of the target analytes decreased as the volume of EDS1 was increased from 400 μL to 600 μL, probably due to the dilution effect of more extractant solvent for the target analytes [36]. Considering the high extraction efficiency, 400 μL was chosen as the optimal volume of the extraction solvent. 

#### 2.1.5. Adsorption Time

Figure 2e illustrates a noticeable trend in the peak areas of the targets, which initially increased and then decreased as the adsorption time varied from 5 min to 25 min. As the adsorption time increases from 5 to 10 min, the peak area increases because it provides sufficient opportunity for interaction between the analyte and the sorbent [37]. After 10 min, the peak area decreases with increasing time; a possible explanation for this is that excess adsorption times can lead to desorption of macroporous adsorption resin, which reduces adsorption efficiency. An adsorption time of 10 min was chosen for further study in light of these observations.

#### 2.1.6. Desorption Time

According to Figure 2f, the extraction efficiency of catechin increases over time within 5–25 min. Within 5–20 min, the extraction efficiency of epicatechin gradually increases with the resolution time. After 20 min, the extraction efficiency of epicatechin decreases, which may be caused by the re-adsorption of the resolved target compounds by the resin [30]. Therefore, 20 min was selected as the best desorption time.

### 2.2. Analysis of Plackett-Burman Design Result

Two levels (−1, 1) were selected for each factor in the Plackett–Burman design (PBD). On the basis of the optimization of the SPE-DES one-factor experiment, the factors that had an influence on the extractive recovery of the flavanols compounds were the molar ratio of DES1 (A), the DES1 volume (B), the amount of XAD-2 macroporous adsorption resin (C), the adsorption time (D), and the desorption time (E). The Plackett–Burman design generated 12 tests to be performed experimentally, as described in Section 3.4: DES-SPE procedure. The experimental design and the results obtained are shown in Table 1 and Table 2.

**Figure 3:**
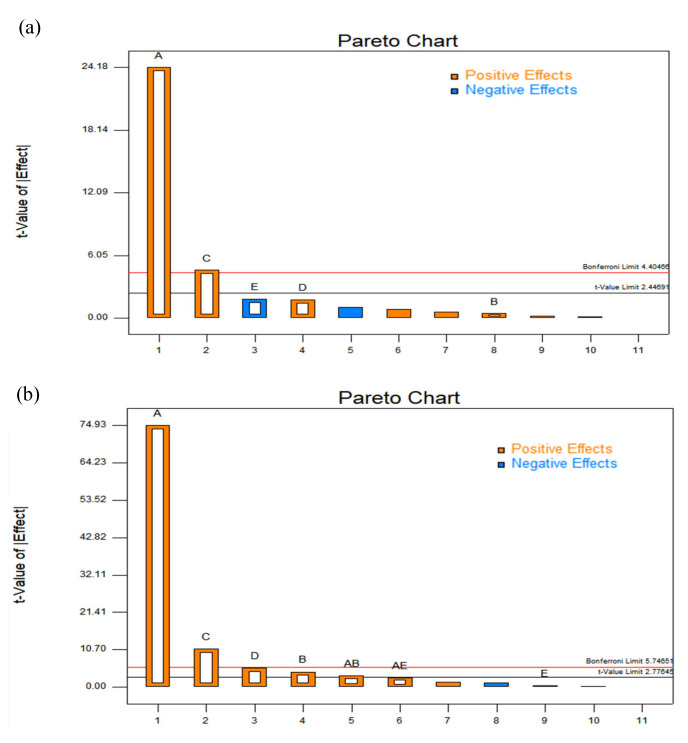
Pareto chart of the standardized effects in the screening. (**a**) Catechin. (**b**) Epicatechin.

Figure 3 shows the standardized effect of each variable on catechin and epicatechin extraction. The bars extending beyond the vertical line correspond to the effects statistically significant at a 95% confidence level. All the significant factors showed a positive regression coefficient value, indicating that the flavanols response increased with an increasing molar ratio of HBA/HBD, the amount of the adsorption resin XAD-2, and the adsorption time. In this figure, the molar ratio of HBA/HBD, the amount of the adsorption resin XAD-2, and the adsorption time had a statistically significant effect on the catechin and epicatechin response (*p* < 0.05), and the order of significance was A > C > D. The remaining factors, including DES1 volume (400 µL) and desorption time (20 min), were kept at a constant value for the following experiments.

### 2.3. Analysis of Box–Behnken Design Results

Because the results of the molar ratio of DES1 (A), the amount of the adsorption resin XAD-2 (B), and adsorption time (C) in the PBD experiment indicated a significant influence, these three factors were optimized and their interactions were analyzed. The BBD-RSM experiments with three factors and three levels were used for the optimization of the experimental conditions (Table 3). The experiments of the 17 runs and the obtained results are shown in Table 4. The peak areas of catechin (Y1) and epicatechin (Y2) were used as the experimental response.

The following second-order polynomial equations obtained from the Design-Expert software analysis were used to express the relationship between the peak areas (Y1 and Y2) and the variables:

Y1 = 271.16 − 12.84A − 8.49B + 1.55C + 3.72AB − 5.95AC + 2.20BC − 19.39A^2^ − 43.44B^2^ − 14.07C^2^

Y2 = 143.80 − 7.96A − 3.45B + 1.61C + 1.50AB + 0.93AC − 2.55BC − 5.49A^2^ − 17.56B^2^ − 4.14C^2^

The *p*-value was used to determine the significance of each coefficient. The *p*-value of the model (*p* < 0.05) implied that the model was significant, and the *p*-value of the lack of fit model (*p* > 0.05) implied that it was not significant relative to the pure error [38]. The ANOVA results for the quadratic polynomial model are shown in Table 5. The *p*-value for both compounds was significant based on the ANOVA results. The lack of fit of catechin and epicatechin was 0.9183 and 0.1237, respectively, being greater than 0.05, indicating that the fitted quadratic model was statistically reasonable and reliable. The coefficient of determination R^2^ of catechin and epicatechin was 0.9832 and 0.9469, respectively, both being greater than 90%, indicating that the experimental data were in high agreement with the predicted extraction results. The variance between R^2^ and R^2^adj of catechin and epicatechin was 0.0215 and 0.0684, respectively, being smaller than 0.2, indicating that the quadratic model fits the actual situation well. The result of the ANOVA show that model can be used to optimize the process of extracting flavanols.

### 2.4. Analysis of 3D Surface Diagram

The 3D surface plots reflect the influence of each independent variable on the response value and also explain the interaction between independent variables [39]. In the 3D response surfaces, the slope of the surface represents the degree of influence of the two variables on the response value—the larger the slope, the steeper the slope, indicating a more significant interaction between the two variables. The contour plot is the bottom projection of the response surface, and if the contour plot tends to be elliptical, it indicates that the interaction between the two factors is significant [40,41]. 

**Figure 4:**
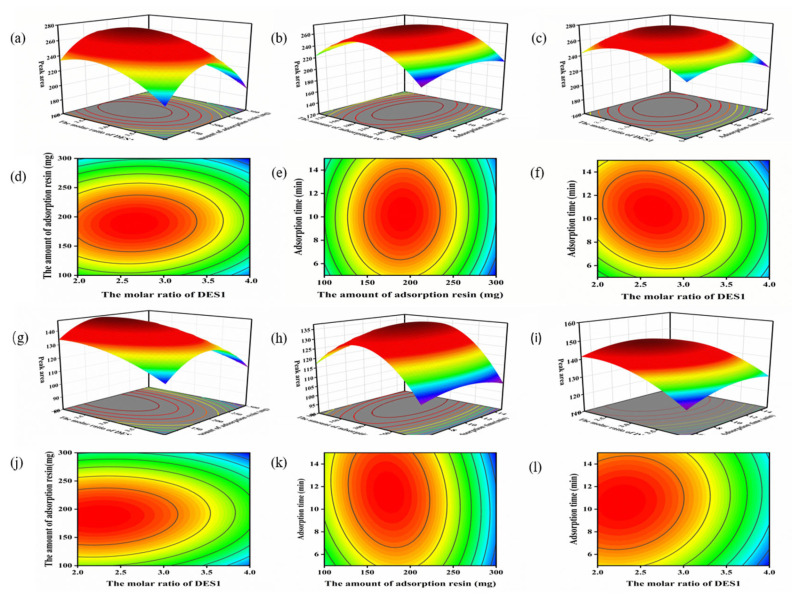
Three-dimensional response surface plots and two-dimensional contour plots of HDES-SPE. (**a**–**l**) indicate the interactions among the molar ratio of HDES, the amount of the adsorption resin XAD-2, and the adsorption time, respectively.

The effects of the molar ratio of DES1, the amount of the adsorption resin XAD-2, and the adsorption time on the peak areas of catechin and epicatechin and their interactions are shown in Figure 4. Figure 4a,g show the effects of the molar ratio of DES and the amount of the adsorption resin XAD-2 on the extraction rate of the two flavanols. When the amount of the adsorption resin XAD-2 was maintained, the extraction rate of the two flavanols increased with the increase in the molar ratio of DES. However, when the molar ratio of DES exceeded a certain value, the extraction rate of the two flavanols increased only slightly, or even declined. Combined with the contour plots, it can be seen that the effect of the molar fraction of DES on the extraction rate of the two target compounds is greater than that of the amount of XAD-2 adsorption resin (Figure 4d,j). Figure 4b,h shows the effects of the amount of the adsorption resin XAD-2 and the adsorption time on extraction rate of the two flavanols. Based on the three-dimensional surface map of the interaction between B and C, the effect of the amount of the adsorption resin XAD-2 on the extraction efficiency was more significant than the adsorption time. It was found that the most important factor affecting the extraction efficiency was the DES1 ratio, followed by the amount of the adsorption resin XAD-2 and the adsorption time.

### 2.5. Validation of Prediction Model

**Figure 5:**
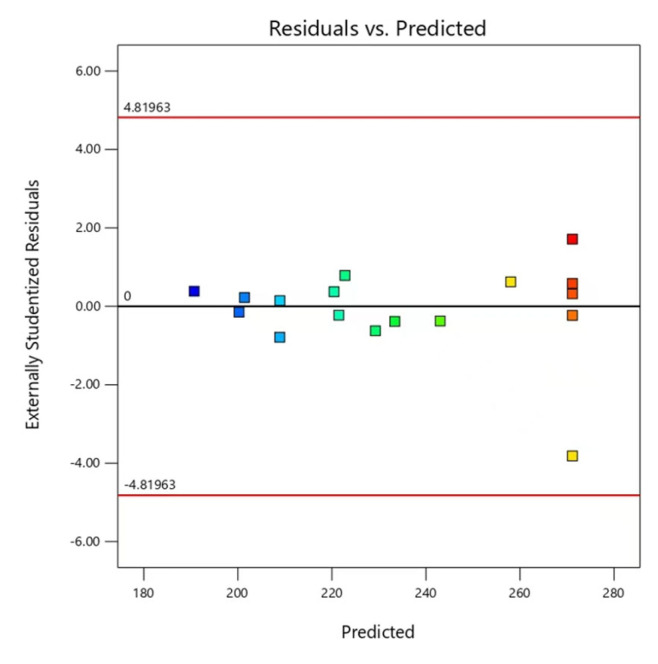
Normal plot of the residual.

**Figure 6:**
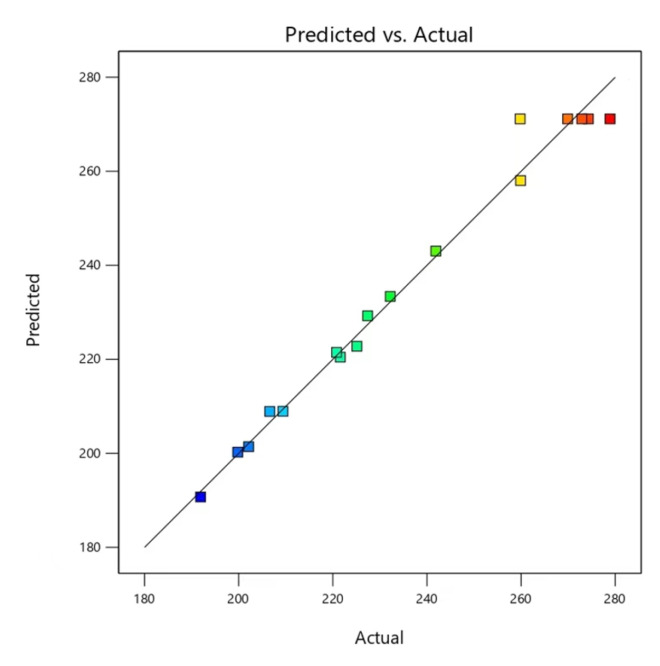
Relationship between the residual and predicted response.

**Figure 7:**
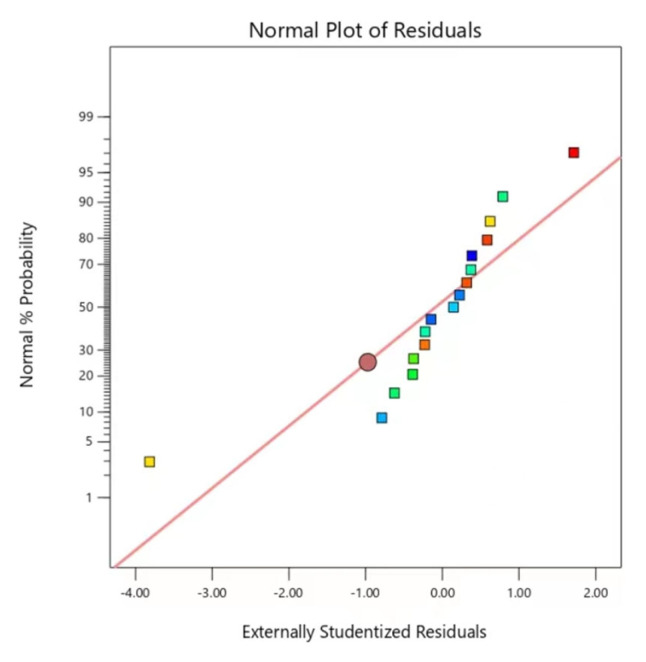
Relationship between the predicted and actual response.

The fitted model needs to be checked to ensure that it adequately approximates the actual situation. If the model shows an inadequate fit, the response surface needs to be further adjusted and optimized to achieve a proper fit [41]. We tested for normality falsity by constructing normality plots of the residual plots, as shown in Figure 5. The normality assumption is satisfied because the points on the residual curve were approximated along a straight line. Figure 6 shows a plot of the residuals against the predicted response of the equation, which was irregularly dispersed on the display, indicating that the model had a good fit to the data. Figure 7 shows the correspondence between the predicted values and the actual values of the test, and since the points were close to the same line, it is shown that the model had a good fit to the data. The results in Figure 5, Figure 6 and Figure 7 are in excellent agreement with the model. Therefore, the predictive model was sufficient for the description of the extraction efficiency of the response surface.

The independent variable regression analysis, 3D surface plot analysis, and normality assumption analysis were performed to predict the optimal extraction conditions using Design-Expert 13.0 software, which were as follows: a molar ratio of DES 1 (A) of 1:2.50; an amount of XAD-2 adsorption resin (B) of 187.73 mg, and an adsorption time (C) of 10.81 min. Combined with the real extrafction process, the factors were adjusted as follows: A = 1:3; B = 188 mg; and C = 11 min.

### 2.6. Method Validation

The linear range, coefficient of determination (R^2^), precision, limit of detection (LOD), limit of quantification (LOQ), and enrichment factor for catechin and epicatechin were determined using the HDES-SPE approach for extraction and HPLC, and the results are given in Table 6. Acceptable linearity was attained in the ranges of 0.5–50 μg/mL for catechin and 0.2–50 μg/mL for epicatechin. The coefficient of determination (R^2^) values was above 0.9917, and the limits of detection (LOD) and quantification (LOQ) were in the ranges of 0.1–0.2 μg/mL and 0.2–0.5 μg/mL, respectively. Relative standard deviations (RSDs) for intra- and inter-day reproducibility ranged from 0.3 to 0.97% and from 0.96 to 4.26%, respectively. The extraction recoveries (ER%) were above 91.3%. These results show that the flavanol extraction procedure developed in the current study had a broad detection range, good stability, and a high sensitivity, and can be used to detect target analytes in true samples.

### 2.7. Analysis of Actual Samples

A higher extraction capacity of the DES means a stronger interaction between the DES and the target compounds in the vinegar samples [35]. In order to evaluate the suitability and accuracy of the method, the extraction and determination of the target compounds were examined using vinegar samples. The standard solutions of flavanol compounds at three concentrations (4, 8, and 25 μg/mL) were added to the actual samples, and the samples were extracted using this procedure and repeated three times for each gradient (Table 7). The recoveries of catechin and epicatechin were in the ranges of 101.3–118.8% and 98.8–108.4%, respectively. The results show that the method had good accuracy. 

According to the optimal extraction conditions, the content of catechin in the five vinegar samples ranged from 0.0544 to 1.0592 mg/mL, and the content of epicatechin ranged from 0.0067 to 0.3360 mg/mL (Table 8). 

### 2.8. Evaluation of Greenness

The main objectives in the field of green analytical chemistry include the miniaturization of sample preparation processes and the employment of green solvents [42]. To quantitatively evaluate the environmental impact of the developed analytical procedure, we utilized the Analytical Eco-Scale framework developed by Gałuszka et al. [43]. This assessment system initiates with a benchmark score of 100 for an ideal analytical method, subtracting Penalty Points (PPs) for each parameter that deviates from the eco-friendly ideal. Methods garnering scores above 75 are considered excellent in terms of greenness, while those exceeding 50 points are deemed acceptable. The PPs are principally calculated based on the quantity of and hazards associated with the chemicals used [42]. Specifically, the number of pictograms under the Globally Harmonized System of Classification and Labelling of Chemicals (GHS) and the corresponding signal words are taken into account. Additionally, the energy consumption of the analytical instrument is assessed. In the present study, the proposed method achieved a score of 71, incurring 29 PPs, as outlined in Table 9, thereby signifying an acceptable level of greenness.

**Figure 8:**
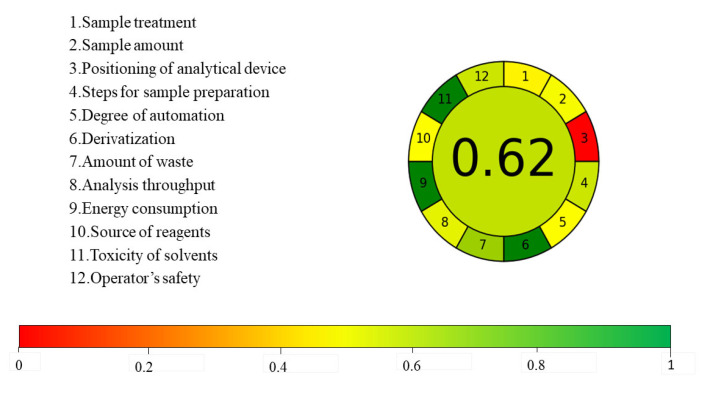
AGREE assessment tool scoring values.

In the pursuit of environmental sustainability within analytical chemistry, we employed the AGREE metric, a comprehensive tool designed to evaluate the greenness of analytical methods. This metric is rooted in the 12 principles of green analytical chemistry and quantifies greenness on a unified scale ranging from 0 to 1. The AGREE framework generates a pictogram that not only reveals the final score but also indicates the performance of the method across different criteria and the weights allocated by the analyst [44]. The developed method achieved an AGREE score of 0.62, as shown in Figure 8. This score is mainly attributed to its excellent performance in principles 6, 9, and 11, which correspond to the absence of derivative agents, no excessive energy consumption, and the use of the least toxic reagents during sample preparation.

### 2.9. Comparative Study of Flavanols Extractions

The proposed SPE-DES-HPLC methodology was compared with previously reported methods for the detection of flavanols in diverse samples, and the results are shown in Table 10. This method required a significantly smaller volume of the extraction solvent and a shorter extraction time compared to the other methodologies. Furthermore, this article outlined a methodology with a low limit of detection (LOD) and a high extraction recovery rate compared to the methods reported in prior studies. It is worth noting that in this study, the DES was subject to little matrix interference in the extraction of the target analytes, and thus can be used as an alternative to toxic organic solvents that is safe for the environment. Therefore, this method is perfectly suitable for detecting and analyzing flavanols in vinegar and has broad application prospects. 

## 3. Experimental

### 3.1. Materials and Reagents

XAD-2 macroporous adsorbent resin was obtained from Duly Biotechnology Co., Ltd. (Nanjing, China). Tetraethylammonium chloride (AR), tetrabutylammonium chloride (AR), choline chloride (AR) and n-caprylate (AR) were obtained from Macklin Biochemical Technology Co., Ltd. (Shanghai, China). Glacial acetic acid was provided by Boruite Chemical Technology Co., Ltd. (Chengdu, China). Phosphoric acid was obtained from Anda Nongsen Technology Co., Ltd. (Shifang, China). Catechin (≥99%) and epicatechin (≥99%) were all obtained from Regal Biology Technology Co., Ltd. (Shanghai, China). Methanol and acetonitrile (HPLC-grade) were purchased from Bruker Co., Ltd. (Beijing, China).

A standard stock solution of 1 mg/mL concentration was obtained by weighing 10 mg of each flavanol standard and dissolving it in 10 mL of chromatographic-grade methanol. A series of standard solutions were prepared by diluting the stock solution into a concentration gradient from 50.00 μg/mL to 0.10 μg/mL.

We added the appropriate amount of macroporous resin into a 25 mL beaker, mixed it with anhydrous ethanol, and allowed it to activate for two hours. Then, we washed it with distilled water until it had no alcoholic flavor, indicating that the activation of the resin was complete.

### 3.2. Instruments and Operating Conditions

The equipment used in this study included the AL204 Analytical Balance by Ditto Biotechnology Co., Ltd. (Shanghai, China) and a high-speed TG16A-W centrifuge from Hunan Saite Xiangyi Centrifuge Instrument Co., Ltd. (Changsha, China). An MX-S adjustable mixer by Da long Xing Chuang Experimental Instrument Co., Ltd. (Beijing, China) and a PH-10 Turbidity Meter from Shanghai Bo Qu Instrument Co., Ltd. (Shanghai, China) were also used. In addition, we employed a solvent filter by Zhejiang NADE Scientific Instrument CO., Ltd. (Hangzhou, China) and a water circulating vacuum pump from Xi’an Morgana Instrument Manufacturing Co., Ltd. (Xian, China).

The chromatographic analyses were performed on the Agilent 1260 HPLC system (Agilent Technologies, Santa Clara, CA, USA) with a C18 reversed-phase column (Waters Technologies, 250 mm × 4.6 mm, 5 µm). The detection wavelength was set to 280 nm, and the injection volume was 10 µL, while the flow rate was 1.0 mL/min. The mobile phase was acetonitrile (phase B)/water containing 0.1% phosphoric acid (phase A). The elution program used the following proportions of solvent A: 0–30 min, 90–65% A; 30–45 min, 65–90% A; 45–55 min, 90% A. The solvents used were all of HPLC grade.

### 3.3. Preparation of Hydrophobic Deep Eutectic Solvent

In this study, several deep eutectic solvents were prepared, including hydrogen bond acceptors (HBA) (tetraethyl ammonium chloride, tetrabutylammonium chloride, choline chloride) and hydrogen bond donors (HBD) (octanoic acid, acetic acid). Homogeneous and transparent deep eutectic mixtures with molar ratios of 1:2, 1:3, 1:4, 1:5, and 1:6 were prepared by means of the heating method at 80 °C.

### 3.4. Solid-Phase Extraction Procedure

**Figure 9:**
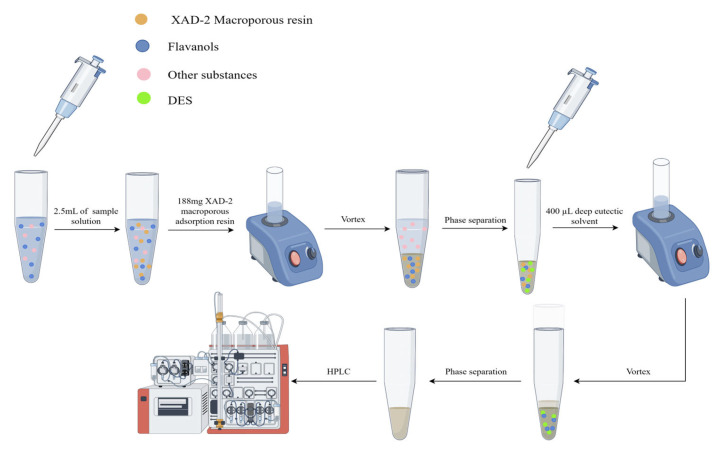
The schematic procedure of the SPE-DES-HPLC.

In the solid-phase extraction procedure, 2.5 mL of the diluted SAV sample solution and 188 mg of XAD-2 macroporous adsorption resin were added to a 5 mL centrifuge tube. The mixture was vortexed via a vortex for 11 min so that XAD-2 could fully adsorb the target. The tube was then centrifuged at 6000 rpm for 5 min and the upper aqueous phase was discarded, and 400 µL of HDES was added to the tube. The vortexing step was repeated for 20 min again to achieve full desorption. After the tube was centrifugated at 6000 rpm for 5 min, the lower aqueous phase was extracted using a syringe and discarded, while the organic phase was collected and filtered through a 0.22 µm organic filter membrane and the resulting organic solution was analyzed by HPLC. The SPE-HDES-HPLC procedure is shown in Figure 9.

### 3.5. Real Sample Preparation

Five Shanxi aged vinegar samples (V-1, V-2, V-3, V-4, V-5) were purchased from a supermarket on Chang Feng Street, Taiyuan, Shanxi Province. Dilution of the various vinegar samples was performed with distilled water. Follow-up samples were processed according to the HDES-SPE procedure.

### 3.6. Calculations of Recovery

The enrichment factor (EF) and extraction recovery (ER%) of the overall HDES-SPE procedure are expressed in Equations (1) and (2), where C0 and C1 refer to the amounts of the two flavanols (catechin, epicatechin) in the initial phase and in the final phase, respectively. Additionally, V1 is the volume of the organic phase and V0 is the volume of the aqueous sample.

(1) $$\mathrm{EF}=\frac{C1}{C2}$$

(2) $$\mathrm{ER}\%=100\times \mathrm{EF}\times \frac{V1}{V0}$$

Relative recovery (RR%) demonstrates the accuracy of the HDES-SPE method. RR% was calculated with Equation (3) for spiked samples containing 4, 8, and 25 μg/mL of each analyte in the DES solution.

(3) $$\mathrm{RR}\%=100\times \frac{\mathrm{Ca}-\mathrm{Cd}}{\mathrm{Ce}}$$

In this equation, Ca is the concentration of the analyte after the addition of a known amount of the same to the original sample solution, Cd is the concentration of the analyte in the original sample solution, and Ce is the concentration of the analyte in a blank solution having the same volume as the one containing the original sample. 

### 3.7. Statistical Analysis

All analytical experiments were analyzed through ANOVA using IBM SPSS Statistics 26 software. The results are presented as the means ± SD of three replicates. The individual and interrelated influences of significant factors on the extraction yield were examined by plotting three-dimensional response surface plots and contour plots through Design-Expert version 12 software and Origin 2019 software. 

## 4. Conclusions

In this study, macroporous resin sorbents and DES solvent were successfully used to extract flavanols from SAV samples. The analysis employed a hydrophobic deep eutectic solvent (HDES) as an environmentally friendly extraction medium during the sample processing stage, and among the types of elution solvents, DES1 (tetraethylammonium chloride and octanoic acid, 1:3) had the highest extraction efficiency. The parameters of the DES-SPE-HPLC method were optimized via single-factor optimization, Plackett–Burman design (PBD) and Box–Behnken design (BBD), resulting in optimal extraction conditions. The proposed SPE-DES-HPLC method provided excellent linearity, a low LOD and LOQ, reliable precision values, and acceptable relative recoveries for the determination of flavanols in Shanxi aged vinegar under optimized parameters. In addition, the greenness of the developed method was assessed by the Analytical Eco-Scale and AGREE, both of which showed that this method is very environmentally friendly. This method is simple, rapid, can be used for the rapid detection of flavanols in vinegar, and can be extended to the extraction of flavanols from other natural products.

## Figures and Tables

**Table 1:** The design matrix of the Plackett–Burman design.

Factor	Level	
	−1	1
The molar ratio of DES1 (A)	1:2	1:4
The volume of DES1 (B)	300	500
The amount of XAD-2 (C)	100	300
Adsorption time (D)	5	15
Desorption time (E)	15	25

**Table 2:** Plackett–Burman design (PBD) with responses of the dependent variables to extraction conditions.

Runs	A	B	C	D	E	Y_1_	Y_2_
1	1:2	500	300	15	15	186.6	112.6
2	1:2	300	300	5	25	169.9	101.5
3	1:4	500	100	5	15	248.5	178.4
4	1:4	300	300	15	25	261.5	192.4
5	1:2	500	100	15	25	155.1	93.9
6	1:2	500	300	5	25	172.5	103.9
7	1:2	300	100	15	15	161.2	98.1
8	1:4	500	300	5	15	258.7	188.2
9	1:4	300	100	5	25	241.2	169.9
10	1:2	300	100	5	15	158.4	90.1
11	1:4	500	100	15	25	252.9	187.6
12	1:4	300	300	15	15	263.9	188.1

Y_1_: catechin peak area (mAu). Y_2_: epicatechin peak area (mAu).

**Table 3:** Factors and levels used in the response surface design.

Factors	Levels		
	Low (−1)	Med (0)	High (+1)
The molar ratio of DES1 (A)	2	3	4
The amount of XAD-2 (B)	100	200	300
Adsorption time (C)	5	10	1

**Table 4:** Results of the BBD for the extraction rates of catechin and epicatechin.

Runs	A	B	C	Y_1_	Y_2_
1	3	200	10	274.3	144.3
2	3	200	10	269.9	142.8
3	2	200	15	259.9	138.1
4	4	200	15	221.6	127.9
5	3	200	10	278.9	147.9
6	3	300	5	202.1	116.6
7	4	200	5	227.4	128.4
8	4	300	10	191.9	109.1
9	3	100	15	220.8	132.7
10	3	300	15	206.6	120.3
11	2	100	10	232.2	135.4
12	2	300	10	209.4	125.9
13	4	100	10	199.8	112.6
14	2	200	5	241.9	142.3
15	3	100	5	225.1	118.8
16	3	200	10	259.8	139.8
17	3	200	10	272.9	144.2

**Table 5:** ANOVA results obtained via the Box–Behnken design.

Source	Epicatechin		Catechin		
	F-Value	*p*-Value	F-Value	*p*-Value	
Model	13.86	0.0011	45.65	<0.0001	significant
A	28.02	0.0011	40.52	0.0004	
B	5.26	0.0555	17.71	0.0040	
C	1.15	0.3193	0.59	0.4673	
AB	0.50	0.5036	1.71	0.2328	
AC	0.19	0.6768	4.35	0.0754	
BC	1.44	0.2697	0.60	0.4657	
A^2^	7.00	0.0331	48.66	0.0002	
B^2^	71.73	<0.0001	244.22	<0.0001	
C^2^	3.98	0.0862	25.61	0.0015	
Lack of fit	3.60	0.1237	0.16	0.9183	not significant
R^2^	0.9469		0.9832		
Adjusted R^2^	0.8785		0.9617		

**Table 6:** Analytical performance of the HDES-SPE-HPLC method in the determination of flavanols.

Analytes	LR ^a^	Standard Curve	R^2 b^	LOD ^c^	LOQ ^d^	EF ^e^	ER ^f^ (%)	RSD ^g^ (%)	
								Intra-Day	Inter-Day
catechin	0.5–50	y = 26.747x − 9.338	0.9917	0.2	0.5	31.0	91.3 ± 0.2	0.30	0.96
epicatechin	0.2–50	y = 73.919x − 37.145	0.9928	0.1	0.2	33.6	98.9 ± 0.3	0.97	4.26

^a^ Linear range (μg/mL). ^b^ Correlation coefficients (R2). ^c^ Limit of detection (μg/mL). ^d^ Limit of quantitation (μg/mL). ^e^ Enrichment factor. ^f^ extraction recoveries. ^g^ Relative standard deviation.

**Table 7:** Results of the recovery rate of catechin and epicatechin.

Sample	Found in Sample (μg/mL)	Added (μg/mL)	Found (μg/mL)	Recovery (%)
catechin	0	4	4.6	115.0
		8	8.1	101.3
		25	29.7	118.8
epicatechin	0	4	4.2	105.7
		8	7.9	98.8
		25	27.1	108.4

**Table 8:** Determination results of flavanols in 5 kinds of Shanxi aged vinegar.

Sample	Catechin/mg·mL^−1^	Epicatechin/mg·mL^−1^
V-1	0.0544	0.0067
V-2	0.0980	0.0742
V-3	0.1062	0.1056
V-4	0.2496	0.1248
V-5	1.0592	0.3360

**Table 9:** The greenness profile of the proposed method using the eco-scale tool.

Items			Penalty Points (PPs)
1. Reagent			
Tetraethylammonium chloride	Amount	<10 mL	1
	Hazard type	Signal word: warning	1
	Hazard amount	1 pictogram	1
Total PPs = 1			
n-octanoic acid	Amount	<10 mL	1
	Hazard type	Signal word: danger	2
	Hazard amount	1 pictogram	1
Total PPs = 2			
Methanol	Amount	10–100 mL	2
	Hazard type	Signal word: danger	2
	Hazard amount	3 pictograms	3
Total PPs = 12			
Phosphoric acid	Amount	<10 mL	1
	Hazard type	Signal word: danger	2
	Hazard amount	1 pictogram	1
			Total PPs = 2
	Amount	<10 mL	1
Acetonitrile	Hazard type	Signal word: danger	2
	Hazard amount	2 pictograms	2
			Total PPs = 4
2. Instruments			
2.1. Energy (kW/h per sample)	HPLC	≤0.1 kWh per sample	0
2.2. Occupational hazard		Analytical process hermetization	0
3. Waste			
3.1. Waste amount		>10 mL	5
3.2. Waste treatment		No treatment	3
			Total PPs = 8
Total penalty points = 29			
Eco-scale score			100 − 29 = 71

**Table 10:** Comparison of the present method with other methods reported for the determination of flavanols.

Method	Analyte	Sample	LOD	LR	Extraction Solvent/	Extraction Time (min)	Sample Volume (mL)	RR (%)	Ref
DLLME-HPLC	Catechin, epicatechin	Wine	0.003–0.114 mg/L	0.192–140.0 mg/L	500 μL ethyl acetate	5 min	4 mL	77.11–113.6%	[45]
MMSPD-UHPLC	Catechin, epicatechin	Radix polygoni multiflori	≤32.24 μL/mL	-	25 mg silica	42 min	25 mg	90.0–100%	[46]
LLME-LC-ESI-MS/MS	Epicatechin	Human milk	74 ng/mL	300–1000 ng/mL	550 µL ethyl acetate	5 min	3 mL	96.8–97.6%	[47]
MSPE-HPLC	Catechin	Green tea	36.1–20.2 mg/L	-	Fe_3_O_4_@MoS_2_@DES-MIP/Methanol-acetic acid	20 min	1 g	98%	[48]
SPE-DES	Catechin, epicatechin	Shanxi aged vinegar	0.1~0.2 μg/mL	0.20–50.00 µg/mL	188 mg XAD-2 macroporous adsorbent resin/DES	36 min	2.5 mL	98.8~118.8%	This work

## Data Availability

All data, tables, and figures are originals.
